# Cardiometabolic Aging Driven by Multi-Organ Crosstalk: Mechanisms and Therapeutic Strategies

**DOI:** 10.3390/ijms27156881

**Published:** 2026-08-01

**Authors:** Shouyao Zhang, Chenggui Xu, Yongli Song, Xinghe Zhang

**Affiliations:** 1 The First Clinical Medical School, Yunnan University of Chinese Medicine, Kunming 650500, China; 13988820865@163.com; 2 The Second Clinical Medical School, Yunnan University of Chinese Medicine, Kunming 650500, China; 18881042806@163.com; 3 School of Basic Medicine, Yunnan University of Chinese Medicine, Kunming 650500, China

**Keywords:** multi-organ, crosstalk, cardiometabolic aging, systemic senescence, therapeutic opportunities

## Abstract

Cardiac senescence is not an isolated organ decline but a systemic consequence driven by pathological crosstalk between the heart and its peripheral metabolic organs. In this review, we discard the traditional organ–centric perspective and construct an integrated framework around multi-organ crosstalk axes, including the epicardial adipose tissue–heart axis, the skeletal muscle–heart axis, the gut–heart axis, and the kidney–heart axis. For each axis, we dissect the local molecular mediators—inflammatory cytokines, lipotoxic metabolites, microbiota-derived compounds such as trimethylamine N-oxide (TMAO), renin-angiotensin-aldosterone system (RAAS) effectors, and extracellular vesicle (EV) cargoes—and illustrate how they converge onto common pathways of oxidative stress, impaired autophagy, and cellular senescence. Importantly, we emphasize that these signals do not operate in isolation; they act synergistically through the circulation, converting local organ dysfunction into systemic cardiac aging via convergence onto shared senescence pathways. By redefining aging as a potentially modifiable multi-organ crosstalk, we propose emerging nodal points—senolytics, myokine mimetics, gut microbiota modulation, RAAS/sodium–glucose cotransporter 2 (SGLT2) inhibitors, and integrated lifestyle strategies—to block pathological crosstalk and delay cardiovascular aging. This framework shifts the research focus from isolated organs to systemic multi-organ crosstalk, providing new insights into cardiometabolic aging.

## 1. Introduction

With advances in medical technology, public health, and socioeconomic development, the global aging population is growing rapidly. By 2050, people aged 65 years and older are expected to account for more than 20% of the US population and more than 29% of the European population [1]. Cardiovascular metabolic aging is inevitably triggered. According to statistics, cardiovascular disease (CVD) has become the leading cause of global disease burden and the main cause of mortality worldwide [2]. CVDs, including hypertension, will affect more than 184 million adults, and the prevalence of hypercholesterolemia, obesity, and diabetes will also increase significantly [3]. Traditional perspectives view cardiovascular aging as an isolated process confined to the cardiovascular system, yet this perspective struggles to explain the increasingly common coexistence of multiple metabolic diseases in clinical settings. Against this backdrop, the American Heart Association (AHA) officially proposed the concept of cardiovascular-renal-metabolic (CKM) syndrome in 2023 [4]. CKM syndrome integrates metabolic risk, chronic kidney disease (CKD), and cardiovascular diseases into a unified management framework [5], suggesting the pivotal role of multi-organ crosstalk in cardiometabolic aging.

Recent biological evidence has increasingly supported the notion of accelerated inter-organ interactions during aging. The bidirectional dialogue between cardiac and skeletal muscle has been confirmed: skeletal muscle synthesizes and releases abundant extracellular vesicles (EVs), myostatin (e.g., follistatin-like protein 1 (FSTL1), fibronectin type III domain-containing protein 5 (FNDC5)/irisin), and microRNAs (e.g., miR-126) mediating communication between skeletal muscle, the heart, and blood vessels, while heart failure (HF) patients often suffer from skeletal muscle atrophy and exercise intolerance, forming a vicious cycle [6]. Epicardial adipose tissue (EAT), as a metabolically active fat depot surrounding the heart, induces myocardial lipotoxicity, fibrosis, and microvascular dysfunction by secreting inflammatory cytokines and free fatty acids, becoming a central node linking obesity to heart failure with preserved ejection fraction (HFpEF) [7,8]. The gut microbiota establishes a bidirectional gut–heart axis through its metabolites, including pro-inflammatory trimethylamine N-oxide (TMAO), phenylacetylglutamine (PAGln), lipopolysaccharide (LPS), protective short-chain fatty acids (SCFAs), hydrogen sulfide (H_2_S), etc., accelerating HF progression [9,10].

Despite the growing recognition of multi-organ crosstalk in cardiometabolic aging, several critical gaps remain: (i) the fragmented nature of existing studies, which typically focus on a single organ axis; (ii) the lack of a unifying framework that integrates molecular mechanisms across different organ systems; and (iii) the insufficient translation of mechanistic insights into axis-specific therapeutic strategies. Our review addresses these gaps by proposing an integrated framework centered on four core axes: adipose tissue–heart, skeletal muscle–heart, gut–heart, and kidney–heart, and systematically linking molecular mediators to shared senescence pathways and potential intervention nodes. They represent the most extensively studied and clinically validated organ systems in the context of cardiometabolic aging and aligning with the CKM syndrome framework (AHA 2023). While we acknowledge that other systems (e.g., liver, immune system, CNS) also contribute, they are beyond the scope of this review; future work should integrate these additional axes into the multi-organ crosstalk paradigm (Table 1).

In this review, we define “cardiometabolic aging” as the progressive, multi-organ decline in metabolic and cardiovascular homeostasis that occurs with advancing age, encompassing subclinical functional deterioration, increased vulnerability to stressors, and the accelerated trajectory toward clinically overt diseases such as HF, CKD, and sarcopenia. We distinguish this from physiological aging (the chronological and biological aging process per se), from end-stage disease states (e.g., established HF with reduced ejection fraction (HFrEF) or end-stage CKD, and from obesity-related cardiomyopathy, which may occur independently of age. While we discuss these disease states as outcomes of accelerated cardiometabolic aging, they are not considered equivalent to the aging process itself.

Based on the above research clues, this review proposes a core argument: cardiometabolic aging is not a passive, organ-autonomous decline, but a cascade reaction driven by multi-organ metabolic crosstalk; pathological changes in any organ can transmit signals to other organs via humoral factors, metabolites, and EVs, ultimately triggering cardiac aging. Therefore, we construct an integrated framework around organ crosstalk axes and further propose actionable intervention nodes, aiming to shift the research focus of cardiac aging from repairing the aged heart to repairing the aged multi-organ crosstalk network.

### Literature Search Strategy

This review is a narrative review. To ensure transparency and reduce concerns regarding selective citation, we performed a systematic literature search in PubMed and Web of Science from database inception to the present, with emphasis on publications from 2021 to 2026. Principal keywords included “cardiometabolic aging,” “multi-organ crosstalk,” “epicardial adipose tissue,” “skeletal muscle myokines,” “gut-heart axis,” “kidney-heart axis,” “senescence,” “inflammaging,” “sodium-glucose cotransporter 2 inhibitors (SGLT-2i),” “glucagon-like peptide-1 receptor agonists (GLP-1 RAs)” and their combinations using Boolean operators. Only English-language articles were included. We screened titles and abstracts for relevance to the four organ axes, prioritized peer-reviewed original articles, systematic reviews, meta-analyses, and major clinical trials, and manually cross-referenced bibliographies of key reviews to identify additional relevant studies. Preclinical vs. clinical evidence is clearly distinguished throughout the text.

## 2. Multi-Organ Crosstalk Axes Driving Cardiometabolic Aging

### 2.1. Adipose Tissue–Heart Axis

The bidirectional interaction between the heart and adipose tissue is one of the key organ crosstalk axes driving cardiometabolic aging. In this interaction network, EAT, due to its anatomical location adjacent to the myocardium without fascial separation, has been widely regarded as a key link connecting obesity to aging-related CVD, especially HFpEF [11,12]. EAT not only serves as an energy storage depot but also functions as a powerful endocrine organ, regulating adjacent myocardium through paracrine and endocrine pathways [13], triggering inflammation, atrial fibrillation, coronary artery disease, cardiac hypertrophy, HF, and myocardial infarction (MI) [14]. The traditional obesity assessment index, body mass index (BMI), cannot distinguish the distribution difference between subcutaneous and visceral fat, and the latter plays a decisive role in driving cardiometabolic aging. Zhou et al. found that EAT volume showed stronger independent predictive value for diastolic dysfunction and adverse clinical outcomes [7], highlighting the clinical significance of epicardial fat distribution in the assessment of cardiometabolic aging. A recent SCOT-HEART trial showed that in 1770 patients with cardiometabolic disease, EAT volume independently predicted the risk of future MI, independent of traditional cardiovascular risk factors, but this association was not present in patients without cardiometabolic disease [15].

The acceleration of cardiovascular metabolic aging by EAT involves multiple mechanisms, initially characterized by an increase in mechanical pressure load. EAT covers 80% of the heart surface and constitutes 20% of the total heart weight, with the EAT of the right ventricle being three to four times that of the left ventricle [16]. When EAT excessively accumulates and hypertrophies, the external compressive force impedes right ventricular filling and venous return, leading to right ventricular dysfunction and exacerbating cardiac mechanical load at the hemodynamic level [12,17,18]. Second is paracrine inflammation and lipotoxicity. Hypertrophic EAT exhibits a pro-inflammatory secretory profile, releasing interleukin-6 (IL-6), tumor necrosis factor-α (TNF-α), monocyte chemoattractant protein-1 (MCP-1), hypoxia-inducible factor-1 (HIF-1), plasminogen activator inhibitor-1 (PAI-1), and various pro-fibrotic factors, inducing myocardial fibrosis [19,20]. Concurrently, excessive EAT accumulation leads to the massive release of free fatty acids that infiltrate cardiomyocytes [21], triggering endoplasmic reticulum (ER) stress and intracellular calcium homeostasis disruption, and inducing insulin resistance, accelerating the development of metabolic cardiomyopathy [22,23]. Third is EV-mediated fat–heart communication, adding a new molecular dimension to the understanding of cardiometabolic aging. Adipose tissue-derived EVs carry various bioactive molecules, including nucleic acids, lipids, and proteins, and act on the heart and vessel walls via the circulation, thereby regulating the functional status and aging process of target cells [24]. Wang et al. found that EV-derived miR-141-3p directly targets PTEN to promote phosphatidylinositol 3-kinase/protein kinase B (PI3K/Akt) signaling, thereby mediating hepatic glucose homeostasis by regulating hepatic neogenesis and glycogen [25]. Notably, specific knockdown of Rab27b GTPase, which is essential for EV secretion, significantly improves the obesity-related HFpEF phenotype, establishing a central role for EV-mediated fat–heart direct communication in cardiometabolic aging [26]. Modulating the adipose tissue secretome, including EVs, protein factors, lipid mediators, and metabolites, has been considered a new direction for treating HF [27].

**Figure 1:**
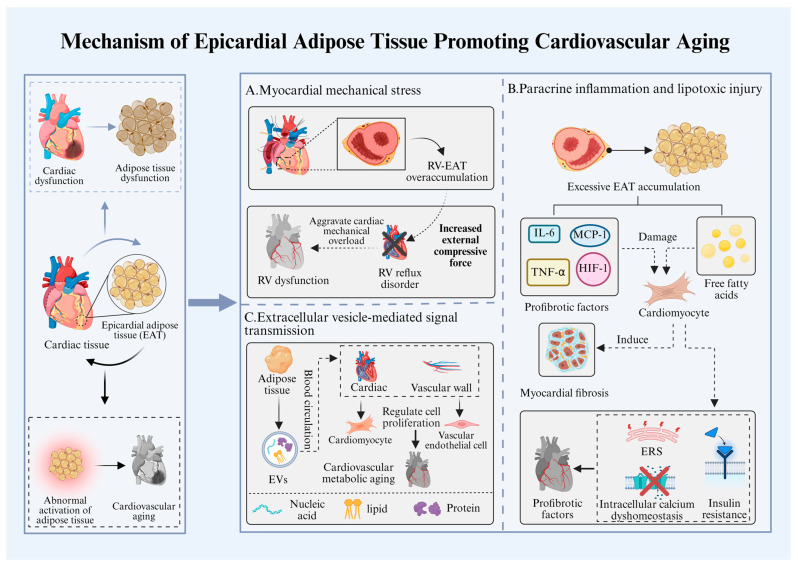
Mechanisms of epicardial adipose tissue in promoting cardiovascular aging. Excessive accumulation and hypertrophy of epicardial adipose tissue may contribute to cardiovascular aging through multiple levels. (**A**) Excessive accumulation of epicardial adipose tissue increases mechanical pressure on the heart, which is associated with hemodynamic disturbances and cardiac dysfunction. (**B**) On one hand, hypertrophic epicardial adipose tissue secretes a large number of inflammatory cytokines and pro-fibrotic factors, suggested to induce myocardial fibrosis; on the other hand, it releases free fatty acids that infiltrate cardiomyocytes, potentially leading to insulin resistance, endoplasmic reticulum (ER) stress, and calcium homeostasis imbalance, thereby possibly exacerbating the development of metabolic cardiomyopathy. (**C**) Adipose tissue-derived exosomal vesicles (EVs) act on the heart and vessel walls through the circulation and target relevant cells, resulting in cardiac aging. Created with BioRender.com, Xu, C. (2026) https://BioRender.com/mx14rjb (accessed on 27 July 2026). Publication license obtained.

Collectively, the adipose tissue–heart crosstalk axis encompasses mechanical pressure, paracrine inflammation, lipotoxic injury, and EV-mediated signal transmission, constituting one of the core hubs driving cardiometabolic aging. These mechanisms are tightly linked to established hallmarks of aging—senescence-associated secretory phenotype (SASP)-mediated inflammaging [28] and mitochondrial dysfunction in EAT all contribute to the progression of this axis [29]. However, whether EAT volume reduction is a direct mediator of cardiovascular benefit or merely a biomarker of metabolic improvement remains an unresolved causal question that warrants further investigation. Future efforts should standardize EAT imaging measurements, deeply analyze the EAT immune–metabolic microenvironment, and conduct large-scale clinical trials directly targeting pathological EAT-myocardial crosstalk, thereby providing precise prevention and treatment strategies for cardiometabolic aging (Figure 1).

### 2.2. Skeletal Muscle–Heart Axis

Emerging evidence emphasizes that skeletal muscle is not only the largest motor organ but also an extremely active endocrine and signal-integrating organ. Skeletal muscle-derived EVs, myokines, and microRNAs mediate inter-organ communication between skeletal muscle and the heart and vessels [6,30], forming the core physiological basis for the protective effects of exercise on the cardiovascular system. Among these, irisin and FSTL1 are the two most intensively studied representative myokines. Exercise upregulates the expression of peroxisome proliferator-activated receptor γ coactivator-1α (PGC-1α) in skeletal muscle, promoting the secretion and release of irisin, a cleaved product of FNDC5 [31]. Irisin further activates AMP-activated protein kinase (AMPK), PI3K/Akt, and other signaling pathways in cardiomyocytes and endothelial cells, exerting protective effects such as improving metabolism, inhibiting inflammation, and alleviating myocardial injury and fibrosis [32,33]. FSTL1 is released in response to resistance exercise after MI and reduces myocardial fibrosis and promotes cardiac angiogenesis via the transforming growth factor-β (TGFβ)-Smad2/3 signaling pathway [34,35]. Notably, exerkines are signaling molecules, including peptides and nucleic acids that released from skeletal muscle (myokines), adipose tissue (adipokines), and other organs in response to exercise, which mediate inter-organ communication [36]. In recent years, Meteorin-like protein (Metrnl), as an emerging exerkine, has received increasing attention for its role in mediating exercise-related cardiovascular benefits [37,38], indicating that skeletal muscle status has a peripheral–central regulatory effect on cardiovascular function.

Likewise, the heart exerts reverse regulation on skeletal muscle, forming a true bidirectional dialogue. In HFpEF models, hyperphosphorylation of skeletal muscle titin is significantly associated with muscle fiber atrophy and reduced muscle strength, accompanied by upregulation of muscle-specific RING finger protein-1 (MuRF1) and abnormal calcium regulatory proteins [39]. Studies have shown that MuRF1 inhibitors can reduce ubiquitin-mediated myofibrillar protein degradation and alleviate HF-related sarcopenia in animal models [40,41]. Moreover, obese HFpEF rats exhibit muscle atrophy, reduced skeletal muscle blood flow, capillary loss, and elevated titin phosphorylation, whereas different HFpEF etiologies exhibit distinct skeletal muscle phenotypes, suggesting that the mechanism of HF-induced skeletal muscle injury may be etiology-specific [42,43]. The clinical consequences of HF-related sarcopenia have been substantiated by ample evidence. Elderly HF patients show decreased skeletal muscle oxidative capacity, accumulation of damaged mitochondria, and excessive mitochondrial reactive oxygen species (mtROS) production, inducing sarcopenia [44,45,46,47], while mitochondria-targeted antioxidants (e.g., MitoQ, SS-31) can clear accumulated mtROS in skeletal muscle and restore mitochondrial bioenergetics [48]. A meta-analysis, including 43 studies with 38,768 HF patients, showed that a low short physical performance battery (SPPB) score was the strongest predictor of poor prognosis in HF, followed by sarcopenia and low muscle strength [49]. Another meta-analysis of 15 studies with 5713 HF patients further confirmed that sarcopenia significantly increased the risk of adverse clinical outcomes by 62%, with all-cause mortality increased by 89% and major adverse cardiovascular events by 37% [50]. These data clearly indicate that HF-driven skeletal muscle injury is an important driver accelerating cardiac disease progression and worsening patient prognosis. In the context of aging, these pathological processes converge on several well-established hallmarks of cellular senescence. Mitochondrial dysfunction and proteostasis loss are particularly prominent in both cardiac and skeletal muscle, where the decline in oxidative capacity and the accumulation of misfolded proteins contribute to contractile dysfunction and atrophy [51,52,53]. Concurrently, impaired nutrient sensing through the AMPK/mTOR pathways further disrupts the metabolic flexibility of these tissues, accelerating age-related functional decline [54].

In summary, the cardiovascular-skeletal muscle crosstalk is a bidirectional, dynamically balanced signaling network. Its forward component mediates the protective effects of exercise on the cardiovascular system via myokines and EVs, while its reverse component mediates skeletal muscle atrophy during HF progression. Maintaining the homeostasis of this axis is crucial for delaying cardiometabolic aging.

### 2.3. Kidney–Heart Axis

There is a deeply coupled bidirectional interaction between the heart and kidneys, with dysfunction of each organ causing and amplifying the other, forming a vicious cycle that accelerates aging and disease progression. The AHA formally proposed the CKM syndrome in 2023. The CKM syndrome integrates metabolic risk, CKD, and CVD into a unified staging management system. Approximately one-third of American adults have at least one CKM risk factor [4,5].

As renal function declines, a large amount of metabolic waste accumulates in the body, forming a pool of uremic toxins such as indoxyl sulfate (IS) and p-cresyl sulfate (PCS) [55]. These toxins activate the aryl hydrocarbon receptor (AhR) and nuclear factor-κB (NF-κB) signaling pathways, inducing inflammatory responses, oxidative stress, mitochondrial dysfunction, and tissue fibrosis in cardiomyocytes, endothelial cells, and fibroblasts, directly impairing myocardial systolic and diastolic function [56]. In CKD patients, characteristic manifestations of cardiac remodeling include left ventricular hypertrophy, myocardial fibrosis, myocardial electrical activity disorders, and capillary rarefaction [57,58]. Notably, a cardiac magnetic resonance imaging study confirmed that imaging and serum biomarkers of myocardial fibrosis (high-sensitivity troponin T, N-terminal pro-B-type natriuretic peptide (NT-proBNP), procollagen type I N-terminal propeptide (P1NP), and procollagen type III N-terminal propeptide (P3NP)) increase with CKD progression, leading to mutual acceleration of renal and cardiac aging processes [59], providing a new dimension beyond hemodynamics for understanding heart–kidney crosstalk.

The long-term consequences of cardiac dysfunction also feed back to damage the kidneys. At the macro-hemodynamic level, reduced cardiac output directly lowers renal perfusion pressure, while systemic venous congestion elevates renal venous pressure, further exacerbating renal interstitial edema and reducing glomerular filtration rate (GFR). At the microcirculatory level, renal medullary hypoperfusion and hypoxia are considered key pathogenic links in HF-related CKD [60]. Indeed, in HF patients, the severity of venous congestion, rather than cardiac dysfunction itself, is the major determinant of worsening renal function. Venous excess ultrasound (VExUS) evidence further reveals that persistent venous congestion impairs glomerular filtration by increasing renal interstitial pressure and reducing trans-renal perfusion pressure [61]. The above hemodynamic disturbances are further compounded by persistently elevated RAAS activity, sympathetic nervous system (SNS) overactivation, and systemic inflammatory responses in HF patients, delivering a multidimensional impact on renal function. Angiotensin II (Ang II) is a potent vasoconstrictor that not only directly impairs glomerular mechanical integrity by elevating intraglomerular pressure and filtration fraction, but also drives tubulointerstitial fibrosis and glomerulosclerosis by stimulating the secretion of pro-fibrotic factors such as transforming growth factor-β1 (TGF-β1), accelerating CKD progression [62,63]. Mitochondrial dysfunction is particularly critical in HF-driven renal injury. The proximal tubule, with its extremely high mitochondrial density, is exquisitely sensitive to mitochondrial DNA (mtDNA) leakage, which further triggers local and systemic inflammatory cascades via activation of cyclic GMP-AMP synthase (cGAS)-stimulator of interferon genes (STING), Toll-like receptor 9 (TLR9), and NOD-like receptor family pyrin domain containing 3 (NLRP3) pathways [64], while excessive mtROS production further aggravates the senescence phenotype and DNA damage of renal tubular cells [65]. These mitochondrial derangements are closely intertwined with other aging hallmarks, including proteostasis loss and cellular senescence [66,67], which collectively drive the progressive functional decline in both organs.

**Figure 2:**
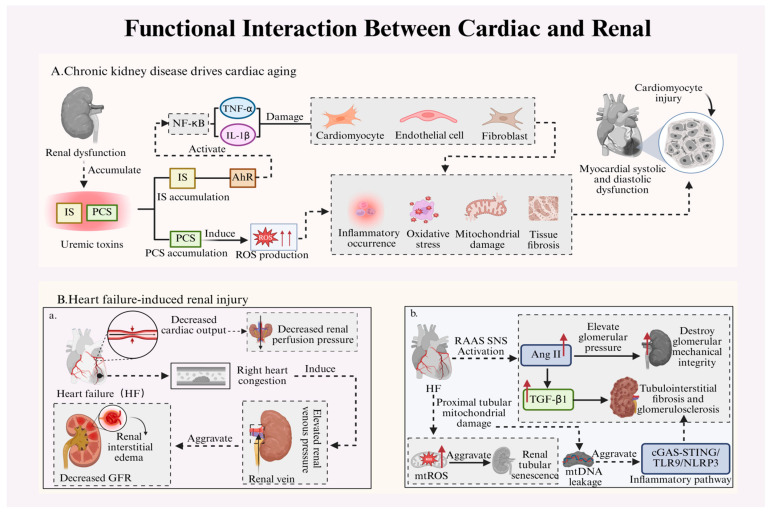
Interplay between the heart and kidneys. The heart and kidneys mutually influence each other: on one hand, renal dysfunction is associated with cardiac aging; on the other hand, heart failure may exacerbate functional impairment in both organs. The red upward arrows in the figure denote elevation and aggravation. (**A**) When renal function declines, a large amount of metabolic waste accumulates in the body, forming a pool of uremic toxins. These accumulated toxins activate the aryl hydrocarbon receptor (AhR) and NF-κB signaling pathways, suggested to induce inflammation, oxidative stress, mitochondrial dysfunction, and tissue fibrosis, potentially leading to cardiac dysfunction. (**B**) Heart failure induces renal function impairment. (**a**) After heart failure, hemodynamic disturbances occur, with reduced cardiac output decreasing renal perfusion pressure. Meanwhile, right heart venous congestion may exacerbate renal interstitial edema, leading to renal dysfunction. (**b**) In heart failure patients, excessive activation of the RAAS and SNS systems is associated with disruption of the mechanical integrity of the glomerulus and stimulates the secretion of pro-fibrotic factors, resulting in glomerulosclerosis. Furthermore, due to the high sensitivity of proximal tubular cells to mitochondria, heart failure-induced mitochondrial dysfunction may activate inflammation-related pathways, further aggravating renal dysfunction. Created with BioRender.com, Xu, C. (2026) https://BioRender.com/59jz3ok (accessed on 27 July 2026). Publication license obtained.

In conclusion, the cardiovascular-kidney crosstalk is a complex bidirectional interaction network driven by hemodynamic factors, RAAS activation, uremic toxins, systemic inflammation, oxidative stress, and other multidimensional factors. A deep understanding of the contribution weight and temporal relationship of each molecular node in this interaction network not only facilitates early identification of high-risk individuals but also provides a theoretical basis for developing novel intervention strategies targeting heart-kidney crosstalk (Figure 2).

### 2.4. Gut–Heart Axis

There is a bidirectional dynamic interaction between the gut microbiota and the cardiovascular system. Pathological gut–heart crosstalk depends not only on the types of metabolites produced by the gut microbiota but also on whether these metabolites can enter the circulation. Under healthy conditions, the intestinal epithelial barrier and gut–vascular barrier effectively restrict bacteria and endotoxins within the gut. However, multiple factors such as aging, a high-fat/high-sugar diet, and a sedentary lifestyle can lead to downregulation of tight junction protein expression, increased epithelial permeability, and elevation of the gut–vascular barrier endothelial marker plasmalemma vesicle-associated protein-1 (PV-1), disrupting the gut barrier and creating a hyperpermeable state [68,69]. This barrier disruption allows large amounts of bacterial endotoxins such as LPS to enter the circulation, becoming a key driver of systemic low-grade chronic inflammation. LPS binds to Toll-like receptor 4 (TLR4) on monocytes and tissue macrophages, activating NF-κB, TLR4, and NLRP3 inflammasome signaling pathways, releasing a cascade of pro-inflammatory cytokines, including interleukin-1β (IL-1β), IL-6, and TNF-α [70], and reducing reverse cholesterol transport (RCT), thereby potentially exacerbating atherosclerosis [71]. TMAO is another representative harmful metabolite during aging. TMAO is produced from dietary choline, carnitine, and other substrates via gut microbiota metabolism [72], and directly promotes oxidative stress, inflammatory responses, and vascular endothelial dysfunction by activating the NLRP3 inflammasome and inhibiting the sirtuin-3 (SIRT3)-superoxide dismutase 2 (SOD2) antioxidant pathway [73]. However, whether TMAO lowering directly improves cardiovascular outcomes or serves primarily as an associative marker of underlying metabolic disturbances remains to be determined. Moreover, gut dysbiosis leads to remodeling of microbiota-derived metabolites, with increased levels of pro-inflammatory, pro-oxidant, pro-fibrotic metabolites, and decreased levels of anti-inflammatory, antioxidant, and cardioprotective metabolites (e.g., SCFAs, H_2_S, indole derivatives) [9]. This shift in metabolite profile constitutes the core molecular mechanism underlying the transition of gut–heart axis function from homeostasis to pathological imbalance.

**Figure 3:**
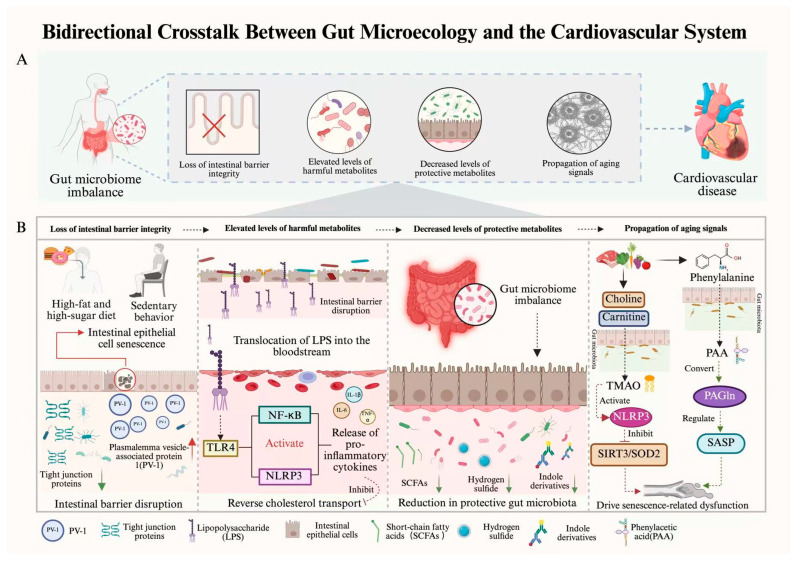
Bidirectional crosstalk exists between the gut microbiome and the cardiovascular system.Red upward arrows denote elevation, and green downward arrows denote reduction. (**A**) Disruption of intestinal barrier integrity, accumulation of detrimental metabolites, depletion of protective metabolites, and transmission of senescence signals contribute to the pathogenesis of cardiovascular diseases. (**B**) Aging, high-fat and high-sugar diets, sedentary lifestyles, and other factors can impair the intestinal barrier, trigger dysbiosis, promote LPS translocation into the bloodstream, activate inflammatory pathways, and induce chronic inflammation that drives atherosclerosis. Dysregulated microbial communities generate TMAO, PAGln, and PAA, which damage blood vessels and myocardium; meanwhile, reduced levels of protective metabolites, along with multiple mechanisms, exacerbate cardiovascular aging and disease progression, increasing the risk of cardiovascular events. Created with BioRender.com, Xu, C. (2026) https://BioRender.com/zj0gvxi (accessed on 27 July 2026). Publication license obtained.

More importantly, during aging, the gut microbiota not only exhibits decreased diversity and compositional imbalance but also establishes novel molecular links with the aging phenotype of cardiovascular cells. Recent studies show that the conversion of dietary phenylalanine to phenylacetic acid (PAA) by the gut microbiota is critical for the production of PAGln, a metabolite associated with atherosclerotic CVD [74]. PAGln drives aortic endothelial cell senescence and impairs angiogenesis via modulation of the SASP [75,76]. PAA directly activates the expression of senescence-associated genes in cardiomyocytes via the novel epigenetic modification of histone phenylacetylation, driving cardiac aging and contractile dysfunction; fecal microbiota transplantation (FMT) experiments confirm that transplanting young gut microbiota reverses this process [77]. Nemet et al., using large-scale untargeted metabolomics, further discovered that PAGln promotes thrombosis and increases cardiovascular event risk by directly activating α2A, α2B, and β2-adrenergic receptors [78]. Thus, PAGln provides a novel pathogenic mechanism for the gut–heart axis that is independent of TMAO-direct regulation of neuroendocrine signaling by metabolites to affect cardiac function. Across this axis, we explicitly connect the molecular mediators to hallmarks of aging, including inflammaging, gut barrier senescence, and epigenetic modifications (Figure 3).

Emerging evidence also links neonatal systemic inflammation to premature cardiac senescence. Lactose intolerance in neonates can trigger systemic inflammatory responses that induce premature cardiac senescence, inflammation, and transcriptome-wide features of oxidative stress [79], suggesting that early-life gut–immune interactions may program long-term cardiovascular aging trajectories [80]. This observation adds a developmental dimension to the gut–heart axis and warrants further investigation.

In summary, pathological gut–heart crosstalk is a cascade process involving the synergistic action of four levels: accumulation of harmful metabolites, reduction of protective metabolites, loss of barrier integrity, and spread of aging signals. Understanding the multidimensional drivers of this axis provides important theoretical support for developing novel intervention strategies targeting the microbiota, inhibiting harmful metabolite production, repairing the gut barrier, and targeting aging-related pathways.

## 3. Therapeutic Strategies Based on Multi-Organ Crosstalk

In this section, we classify interventions according to their therapeutic maturity: (1) established therapies supported by large-scale randomized controlled trials (e.g., GLP-1 RAs, SGLT-2i, RAAS inhibitors); (2) emerging clinical therapies supported by phase III trials (e.g., angiotensin receptor–neprilysin inhibitors (ARNIs), mineralocorticoid receptor antagonists (MRAs); and (3) experimental/preclinical strategies supported primarily by preclinical or early-phase clinical data (e.g., senolytics, MuRF1 inhibitors, engineered EVs, myokine mimetics, FMT).

### 3.1. Adipose–Heart Axis: From Metabolic Inflammation to Targeted Intervention

Given the central role of EAT in driving cardiometabolic aging, targeting EAT has become an important strategy to delay cardiovascular aging. Current interventions targeting the adipose–heart axis mainly include the following categories.

#### 3.1.1. GLP-1 Receptor Agonists

Glucagon-like peptide-1 (GLP-1) is an incretin hormone secreted by intestinal L cells that promotes insulin secretion in a glucose-dependent manner and inhibits glucagon release, and exerts cardiovascular protective effects by binding to GLP-1 receptors (GLP-1R) expressed in cardiac and vascular tissues [81]. Common GLP-1R agonists, such as liraglutide [82] and semaglutide [83], reduce EAT volume and improve its inflammatory status, thereby possibly ameliorating CVD risk in overweight or obese individuals. Exenatide enhances adiponectin (APN) production and promotes fatty acid catabolism, thereby slowing vascular aging and atherosclerotic plaque progression [84]. Moreover, tirzepatide, a dual glucose-dependent insulinotropic polypeptide (GIP)/GLP-1 receptor agonist, not only suppresses appetite and significantly reduces body weight in type 2 diabetes mellitus (T2DM) patients [85] but also reduces total cholesterol, low-density lipoprotein, and triglycerides; decreases EAT; and exerts anti-atherosclerotic and anti-HF effects [86,87]. Dipeptidyl peptidase-4 (DPP-4) is a ubiquitous multifunctional glycoprotease that rapidly degrades GLP-1. DPP-4 inhibitors (e.g., sitagliptin, vildagliptin) delay the degradation of endogenous GLP-1 by inhibiting DPP-4 activity, thereby indirectly enhancing GLP-1 signaling [88]. However, it should be noted that DPP-4 inhibitors have not demonstrated consistent cardiovascular benefit in large outcome trials, and some agents (e.g., saxagliptin) have been associated with a potential HF signal. Thus, their role in targeting the adipose–heart axis remains under investigation.

#### 3.1.2. Sodium-Glucose Cotransporter 2 Inhibitors (SGLT-2i)

SGLT-2 inhibitors are a class of novel antidiabetic drugs that reduce glucose reabsorption and promote urinary glucose excretion by inhibiting SGLT-2 expression in the renal proximal tubule, thus improving glycemic control. More importantly, SGLT-2is have demonstrated cardiovascular protective effects beyond glucose lowering in cardiovascular outcome trials [89]. Mechanistic studies indicate that SGLT-2i reduce inflammatory infiltration and ferroptosis of EAT in HF patients and improve myocardial energy metabolism and diastolic function [90]. Clinical studies likewise show that empagliflozin [91] and dapagliflozin [92] reduce epicardial fat thickness and myocardial glucose uptake in T2DM patients, significantly increasing myocardial flow reserve, in part due to their regulatory effects on EAT. Of note, luseogliflozin, an SGLT2 inhibitor, has been shown to reduce EAT accumulation in patients with T2DM [93]. Therefore, SGLT-2is are regarded as important drugs targeting the fat–heart axis. In addition to the above drugs, other strategies targeting EAT are also being explored. For example, modulating the secretion and function of adipose tissue-derived EVs and their carried microRNAs (e.g., miR-141-3p) may become a new direction to interrupt pathological adipose–heart crosstalk [94]. Future large-scale randomized controlled trials are required to further validate the long-term efficacy and safety of these strategies in delaying cardiometabolic aging.

### 3.2. Skeletal Muscle–Heart Axis: From Exercise Protection to Nutritional Support

#### 3.2.1. Physical Exercise

Physical exercise is the most well-established modulator of the skeletal muscle–heart axis, exerting pleiotropic protective effects on the heart. Numerous studies have shown that aerobic exercises such as running and swimming significantly enhance skeletal muscle antioxidant capacity, upregulate ubiquitin–proteasome pathway-related proteins MuRF-1, Atrogin-1, and cathepsin L, prevent chronic heart failure (CHF)-induced oxidative damage, and increase cardiac index [95,96]. In post-MI HF models, exercise training restores mitochondrial oxygen consumption, reduces H_2_O_2_ release, alleviates myocardial fibrosis and apoptosis, and regulates mitochondrial dynamics [97,98,99]. Regarding the optimal exercise intensity, current evidence is not fully consistent. Moreira et al. suggested that moderate-intensity continuous training (CMT) and high-intensity interval training (HIT) have similar effects on improving cardiac contractile function, skeletal muscle redox balance, and preventing muscle atrophy, but HIT is superior to CMT in improving aerobic capacity [100]. Lu et al. further confirmed that HIT induces higher concentrations of glutathione peroxidase (GPx), phosphofructokinase-1 (PFK-1), and carnitine palmitoyltransferase-1 (CPT-1) than CMT and also increases the proportion of ATP synthesis; thus, HIT may have greater advantages in improving cardiac function and reducing oxidative stress [101]. Meng et al. confirmed that resistance training, CMT, and HIT all improve post-MI cardiac function, reduce cardiac fibrosis, and regulate the expression of insulin-like growth factor-1 (IGF-1), mechano-growth factor (MGF), neuregulin-1 (NRG1), and myostatin (MSTN), activating Akt and extracellular signal-regulated kinase 1/2 (Erk1/2) signaling pathways in the soleus muscle, thereby increasing muscle weight and myofiber cross-sectional area [102]. Although exercise is generally beneficial, there is currently no consensus regarding the optimal exercise intensity. Despite the potential advantages of high-intensity interval training in improving cardiorespiratory function, its long-term safety and applicability to patients with different subtypes of heart failure require further investigation.

#### 3.2.2. Nutritional Support

Nutritional support and metabolic intervention play a key role in restoring skeletal muscle anabolism. Supplementation with essential amino acids (EAAs) has been shown to be an effective therapeutic strategy. In 60 elderly patients with HFrEF and sarcopenia, EAA supplementation significantly improved sarcopenia indicators, markedly reduced oxidative stress biomarkers such as Nox-2 and 8-iso-prostaglandin F2α, and significantly increased left ventricular ejection fraction (LVEF) and global longitudinal strain (GLS) [103]. Intravenous iron therapy focuses on improving skeletal muscle mitochondrial energy metabolism. In addition to intravenous iron supplementation, dietary strategies for iron deficiency compensation should not be overlooked. Folate, abundant in leafy green vegetables and avocado, plays a critical role in hematopoiesis and iron metabolism [104,105]. Therefore, a combined dietary approach that ensures adequate intake of both iron and folate represents a practical and accessible complementary strategy for managing iron deficiency in HF patients, particularly in resource-limited settings. Evangelista et al. conducted a systematic review of 10 randomized controlled trials of high-protein dietary interventions in HF patients, further confirming that high-protein diets increased walking distance, improved muscle strength, and reduced HF rehospitalization rates by 18% [106]. Omega-3 polyunsaturated fatty acids have been widely recognized for their cardiovascular protective value, and their role in promoting skeletal muscle health has received increasing attention. Karimi et al. pointed out that omega-3 fatty acids not only improve muscle strength in healthy individuals but also show positive effects on enhancing large-vessel endothelial function, reducing arterial stiffness, and increasing nitric oxide bioavailability [107]. Creatine, a naturally occurring nitrogen-containing organic acid in skeletal muscle and myocardium, serves as an energy buffer through the phosphocreatine system, facilitating rapid ATP resynthesis, and plays a key supportive role in high-energy-demand tissues such as skeletal muscle and myocardium [108]. The FERRIC-HF II study showed that 2 weeks of ferric derisomaltose treatment significantly enhanced skeletal muscle mitochondrial complex I-dependent respiration in HF patients, providing the first human evidence that iron supplementation improves skeletal muscle function by enhancing mitochondrial electron transport chain activity [109]. These positive findings regarding metabolic and nutritional support provide important clinical translation evidence for breaking HF-driven muscle atrophy.

### 3.3. Kidney–Heart Axis: From Hemodynamics to Multi-Target Drugs

The core of therapeutic strategies targeting kidney–heart crosstalk lies in interrupting the vicious cycle between RAAS overactivation and progressive loss of renal function, while also addressing cardiac structural repair and metabolic homeostasis re-establishment. A shift from traditional “blood pressure lowering-diuresis” to “anti-aging-multi-organ protection” is required.

#### 3.3.1. RAAS Inhibitors

RAAS inhibitors, including angiotensin-converting enzyme inhibitors (ACEIs), angiotensin receptor blockers (ARBs), and MRAs, are first-line drugs for the treatment of HFrEF in appropriately selected patient populations [110]. In HFpEF, the evidence is more modest, and guideline recommendations vary [111]. In CKD, RAASi use should be guided by albuminuria level and baseline renal function [112]. Contemporary guideline-directed medical therapy (GDMT) often includes the addition of SGLT2 inhibitors and MRAs as part of a multi-pillar approach [113]. Patel et al., in a cohort study of 168,860 HF patients, showed that those receiving high-dose RAAS inhibitors had a 15% lower risk of kidney failure within 5 years [114]. Cox proportional hazards model analysis indicated that, for CKD patients with HFrEF, RAS inhibitors also reduced the risk of adverse cardiovascular events and all-cause mortality [115]. MRAs provide irreplaceable cardiorenal protection by blocking aldosterone-dependent and -independent pro-fibrotic signaling pathways. FIDELIO-DKD and FIGARO-DKD phase III trials have demonstrated that non-steroidal MRA finerenone significantly reduces renal and cardiovascular event risks in patients with CKD and T2DM [116,117]. ARNIs are a novel class of drugs that simultaneously act on the functional regulatory mechanisms of the heart and kidneys. Data from the STRATS-HF-ARNI registry showed that in 1074 HF patients receiving sacubitril/valsartan, LVEF improved, and cardiovascular outcomes were superior to those with traditional RAAS inhibitors [118].

#### 3.3.2. SGLT2 Inhibitors

SGLT2 inhibitors, originally developed as antidiabetic drugs, including empagliflozin, dapagliflozin, and canagliflozin, are now widely recognized as foundational drugs for cardiorenal protection, with their protective effects extended from diabetic populations to non-diabetic HF patients and CKD patients [119]. At the kidney level, these drugs significantly reduce HF hospitalizations and CKD progression by ameliorating glomerular hyperfiltration, reducing oxidative stress and inflammation, and optimizing cardiac energy metabolism [120]. Importantly, their cardiorenal protective effects are significant regardless of the patient’s estimated glomerular filtration rate (eGFR) level [121]. At the cardiac level, DAPA-HF and EMPEROR-reduced trials showed that SGLT2 inhibitors significantly reduce HF hospitalizations and cardiovascular death in HFrEF patients, regardless of diabetes status [122,123]. Further, Solomon et al. extended these benefits to patients with HFpEF [124]. Moreover, SGLT2 inhibitors reduce venous congestion via osmotic diuresis and improved vascular compliance, thereby reducing cardiac preload and afterload without activating the neurohormonal system, avoiding the prerenal injury caused by traditional diuretics [125,126]. In addition, SGLT2 inhibitors may inhibit sympathetic overactivity by modulating neurohumoral activation and renal afferent signaling pathways, which is a potential mechanism of cardiorenal protection [127]. These strategies collectively constitute a multi-dimensional intervention network beyond RAAS inhibitors.

### 3.4. Gut–Heart Axis: From Dysbiosis to Microecological Reconstruction

#### 3.4.1. Probiotics and Fecal Microbiota Transplantation

Based on the upstream driver of pathological gut–heart crosstalk–gut dysbiosis, restoring microbial homeostasis through exogenous probiotics or prebiotics constitutes the most direct and fundamental intervention strategy for gut-heart crosstalk. A meta-analysis published in 2025, including 11 studies, confirmed that probiotic intervention mildly improves cardiac function indices (LVEF, left ventricular end-systolic volume); reduces levels of inflammatory factors such as high-sensitivity C-reactive protein (hs-CRP), IL-6, and TNF-α; modulates the proportion of dominant bacterial taxa; and reduces HF readmission rates [128]. Another meta-analysis of HF patients similarly confirmed that probiotic supplementation can serve as an anti-inflammatory, antioxidant, and gut microbiota-modulating agent for HF or MI patients, effectively improving HF-related inflammatory markers and clinical outcomes [129].

FMT is an innovative intervention strategy that transplants the entire gut microbiota from a healthy donor into the patient’s body to reconstruct a functional micro-ecosystem. Compared with selective interventions such as probiotics or prebiotics, FMT can restore the recipient’s full microbial metabolic network in a single intervention, showing greater potential for severe dysbiosis [130,131]. In recent years, multiple animal studies have confirmed that FMT improves HF-related cardiac function and metabolic phenotypes. In a mouse model of MI-reperfusion-induced HF, FMT from mice treated with the Chinese herbal preparation Xin-Ji-Er-Kang (XJEK) partially improved cardiac function in recipient mice [132]. Moreover, transplanting young mouse fecal microbiota into aged mice significantly improved vascular endothelial-dependent diastolic dysfunction and metabolic disorders in aged mice by activating the AMPK/SIRT1 signaling pathway, restoring telomere function, and reducing vascular inflammation [133]. However, it must be emphasized that FMT remains an experimental intervention in cardiovascular disease [134,135]. Major challenges include safety concerns [136], donor selection [137], durability of effect, and regulatory/standardization issues [138]. Large, long-term trials are needed before FMT can be considered for clinical adoption in cardiovascular diseases. FMT research in CVD is moving from exploratory studies to early clinical trials. An AHA science advisory has listed gut microbiome modulation as an emerging direction for hypertension treatment [139]. A multicenter, randomized, double-blind, placebo-controlled trial evaluating FMT for hypertension found that oral healthy microbiota capsules significantly reduced systolic blood pressure compared to placebo after 1 week of intervention, without safety concerns; however, the long-term effect on blood pressure control remains to be clarified [140]. Future efforts should explore multiple FMT courses and engraftment of specific functional bacterial strains to overcome the current bottleneck of transient FMT efficacy, opening new avenues for precision therapy of the gut–heart axis.

#### 3.4.2. Dietary Intervention

**Figure 4:**
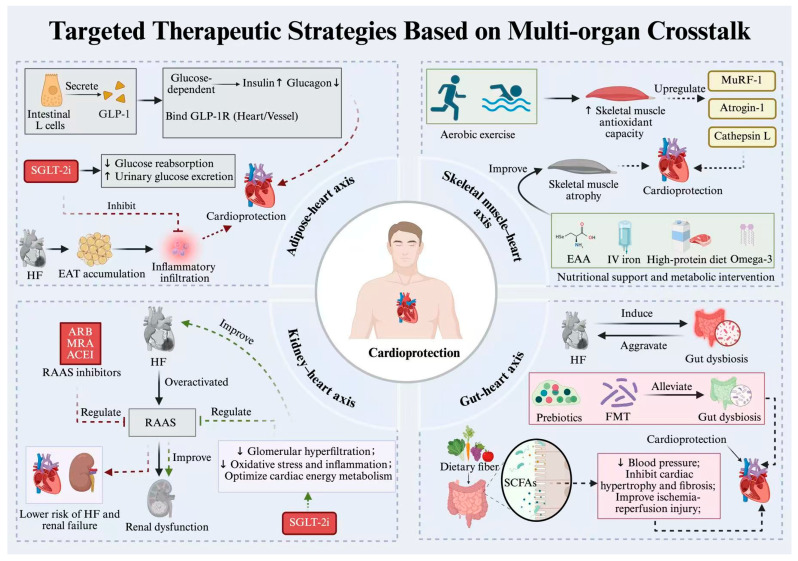
Multi-organ crosstalk-based targeted therapeutic strategies. Black upward arrows in the figure denote elevation, and downward arrows denote reduction. In the setting of heart failure, pathological crosstalk among adipose tissue, skeletal muscle, kidneys, and gut may constitute a core driver of disease progression. Targeting this network is a key direction for comprehensive heart failure management. Adipose tissue: Pharmacological agents such as SGLT2 inhibitors and GLP-1 receptor agonists reduce visceral fat accumulation, may alleviate systemic inflammation and lipotoxicity, and may block adiposity-induced myocardial damage signals. Skeletal muscle: Aerobic exercise strengthens atrophied skeletal muscle, while nutritional support with amino acids and other supplements may ameliorate sarcopenia and restore the secretion of protective myokines, thereby potentially reducing myocardial fibrosis and exerting cardioprotective effects. Kidneys: Targeting the kidney system affected by heart failure with mineralocorticoid receptor antagonists and SGLT2 inhibitors improves renal dysfunction and reduces the incidence of heart failure. Gut: After heart failure, gut microbiota dysbiosis occurs, which can be corrected by probiotic supplementation, fecal microbiota transplantation, or dietary intervention to modulate microbial composition, repair the intestinal barrier, and improve heart failure. Created with BioRender.com, Xu, C. (2026) https://BioRender.com/jcqy78c (accessed on 27 July 2026). Publication license obtained.

Dietary intervention and strategies directly targeting harmful metabolites occupy an important position in gut–heart axis therapy. Dietary fiber is fermented by the gut microbiota to release acidic metabolites known as SCFAs [141]. Studies have found that SCFAs exert cardioprotective effects by lowering blood pressure, inhibiting cardiac hypertrophy and fibrosis, and ameliorating ischemia–reperfusion injury; the number of SCFA-producing bacteria in the gut of HFpEF patients is significantly reduced, exacerbating HF progression [142,143]. A randomized, double-blind trial demonstrated that SCFA supplementation (acetate and butyrate) significantly lowered systolic blood pressure in hypertensive patients [144]. Another study found that dietary fiber supplementation (inulin) reduced traditional cardiovascular risk factors such as cholesterol, triglycerides, and blood pressure, possibly due to increased SCFA abundance [145]. This may be related to the inhibition of harmful metabolites such as TMAO by plant -based dietary patterns [146]. Another animal study found that SCFAs produced by the gut microbiota are crucial for immune function and cardiac repair after MI [147]. Furthermore, food and medicine homologous components such as berberine have been found to reduce intestinal TMA production by binding and inhibiting CutC/D enzyme activity, thereby lowering circulating TMAO levels [148]. Resveratrol (RSV), a natural plant polyphenol with anti-atherosclerotic effects, increases the abundance of Lactobacillus and Bifidobacterium and promotes hepatic bile acid production to alleviate TMAO-induced atherosclerosis [149]. The above studies have accumulated substantial evidence for dietary intervention in reducing cardiovascular event risk, but their clinical efficacy still requires confirmation by large-scale randomized trials (Figure 4).

## 4. Conclusions

Cardiometabolic aging is not a passive decline of the heart as a single organ, but a systemic self-accelerating process driven by pathological crosstalk between the heart and multiple peripheral metabolic organs, including epicardial adipose tissue, skeletal muscle, the gut, and kidneys. This review has focused on four core axes: the adipose–heart axis, skeletal muscle–heart axis, gut–heart axis, and kidney–heart axis, and has outlined the specific molecular mediators of each axis, including inflammatory cytokines, lipotoxic metabolites, TMAO and PAGln, RAAS effectors, and EVs, illustrating how these signals may converge on common pathways of oxidative stress, impaired autophagy, and cellular senescence, converting local organ dysfunction into systemic cardiac aging.

Based on the above mechanistic analysis, we propose axis-specific intervention strategies. For the adipose–heart axis, GLP-1 receptor agonists, SGLT2 inhibitors, and weight loss interventions can reduce EAT volume and improve its inflammatory profile. For the skeletal muscle–heart axis, regular exercise exerts cardiovascular protection by upregulating myokines such as irisin, FSTL1, and Metrnl, with exercise intensity needing individualization; concurrently, nutritional support such as EAAs, intravenous iron, and MuRF1 inhibitors can counteract HF-driven skeletal muscle atrophy. For the gut–heart axis, probiotics, dietary fiber, and FMT may modulate microbiota-derived metabolites, reduce TMAO, and repair the gut barrier. For the kidney–heart axis, RAAS inhibitors, SGLT2 inhibitors, and finerenone constitute a dual-pillar cardiorenal protection strategy. In addition, senolytics and lifestyle interventions may simultaneously suppress multiple axes of crosstalk. However, it should be emphasized that several of these interventions—particularly senolytics, MuRF1 inhibitors, engineered EVs, myokine mimetics, and FMT—remain experimental and are supported primarily by preclinical or early-phase clinical data. Their clinical application requires further validation.

Beyond the organ-specific mediators discussed above, inflammation and inflammasome activation represent a unifying mechanism across multiple crosstalk axes in cardiometabolic aging. The NLRP3 inflammasome, in particular, has emerged as a central driver of cardiac fibrosis in metabolic and arrhythmic disease contexts [150]. Upon activation by metabolic danger signals (e.g., lipotoxicity, oxidative stress, or microbial products), NLRP3 triggers caspase-1-dependent IL-1β and IL-18 release. These inflammatory mediators also promote the activation of fibroblasts, thereby enhancing the production of extracellular matrix (ECM) proteins [151]. This fibrotic response, driven by inflammatory bodies, impairs both diastolic and systolic functions and exacerbates the occurrence of ventricular arrhythmias [152]. Collectively, these observations position the inflammasome as a promising therapeutic target for mitigating both structural and electrical consequences of cardiometabolic aging, although clinical translation remains at an early stage.

It is noteworthy that the developmental origins of health and disease (DOHaD) concept suggests that adverse early-life exposures can epigenetically program the cardiovascular system toward an accelerated aging trajectory [153,154], such as maternal malnutrition and intrauterine growth restriction manifesting as obesity, type 2 diabetes, and cardiovascular diseases in adulthood [155,156]. This developmental dimension adds an important temporal layer to the multi-organ crosstalk framework, suggesting that aging trajectories may be established long before clinical disease emerges.

Growing evidence supports a bidirectional relationship between cardiovascular disease and neuropsychiatric conditions such as anxiety, tension, and depression [157]. Chronic psychological stress and depressive states may contribute to the progression of cardiometabolic aging through shared mechanisms, including sustained hypothalamic-pituitary–adrenal (HPA) axis activation [158]. Conversely, within the patient population suffering from cardiovascular diseases, the prevalence of depression or anxiety disorders is approximately 20.8%. These findings underscore the intimate association between cardiovascular diseases and depressive or anxious symptoms, which, in turn, contributes to an increased all-cause mortality rate [159]. These observations suggest that screening for and managing mental health comorbidities may represent a complementary strategy to slow the progression of cardiometabolic aging, although further interventional studies are needed to confirm this hypothesis.

Future research directions should focus on several key areas. Rather than broad calls for large-scale longitudinal cohort studies, we propose specific, testable research questions for each axis:

(1) Adipose–heart axis: Does pharmacological reduction of EAT volume by GLP-1RAs or SGLT2 inhibitors independently lower cardiac senescence biomarkers in a dose-dependent manner, and is this effect sustained after drug withdrawal?
(2) Skeletal muscle–heart axis: Does long-term (≥1 year) high-intensity interval training upregulate circulating irisin levels sufficiently to reduce myocardial fibrosis, as measured by cardiac MRI, in HFpEF patients?
(3) Gut–heart axis: Does FMT from young donors reduce circulating PAGln and TMAO levels in elderly HF patients by ≥30%, and is this reduction associated with improved cardiac diastolic function?
(4) Kidney–heart axis: Does finerenone add to the protective effects of SGLT2 inhibitors on the combined endpoint of cardiac and renal senescence markers in patients with CKM syndrome, and is this effect independent of blood pressure lowering?

Second, explore the synergistic effects of axis-specific interventions with broad-spectrum anti-aging drugs, for example, head-to-head comparison trials of SGLT2 inhibitors combined with finerenone or senolytics in CKM syndrome. Third, promote the clinical translation of novel therapies such as FMT, engineered EVs, and myokine mimetics, focusing on bottlenecks such as engraftment efficiency, targeted delivery, and long-term safety. Fourth, integrate the multi-organ crosstalk framework into the current precision staging and management pathways of cardiometabolic diseases, achieving a paradigm shift from single-organ treatment to systemic network intervention. Through these efforts, cardiometabolic aging may be transformed from an irreversible physiological process into a targetable, intervenable, and delayable systemic syndrome.

## Figures and Tables

**Table 1:** Summary of multi-organ crosstalk axes in cardiometabolic aging.

Organ Axis	Primary Mediator	Major Cardiac Consequences	Evidence Level	Candidate Biomarkers	Treatment Maturity
Adipose tissue–cardiovascular	Pro-inflammatory paracrine factors	Myocardial fibrosis	High (multiple clinical cohort studies, basic mechanistic research)	Interleukin-6 (IL-6), tumor necrosis factor-α(TNF-α), monocyte chemoattractant protein-1 (MCP-1), plasminogen activator inhibitor-1 (PAI-1)	Medium (anti-inflammatory and anti-fibrotic drugs are currently under investigation)
	Mechanical effects	Right ventricular dysfunction	Medium (human anatomy and hemodynamic observational studies)	Cardiac Imaging: epicardial adipose tissue (EAT) volume, EAT thickness	Low (no targeted interventions available; used only as an imaging-based risk assessment marker)
	Lipotoxic mediators	Metabolic cardiomyopathy	Medium (basic mechanistic research)	Circulating free fatty acids, myocardial lipid deposition imaging markers	Medium (lipid metabolism-improving drugs may indirectly intervene)
	Extracellular vesicle (EV)-mediated signaling	Regulation of myocardial signaling pathways, promoting obesity-related HFpEF, and involvement in glucose–lipid metabolic disorders and myocardial aging	Medium (basic mechanistic research)	Circulating fat-derived EVs, EV-encapsulated miR-141-3p, Rab27b expression levels	Low (in basic research stage; no clinical treatment options yet)
Skeletal Muscle–Cardiovascular	Exercise factor (Irisin, EVs, FSTL1)	Cardioprotective effects	Medium (basic mechanistic research)	Various Myokines and Skeletal Muscle-Derived Extracellular Vesicles (EVs)	Low (in basic research stage; no clinical treatment options yet)
Kidney–Cardiovascular	Uremic toxins	Impairment of myocardial contractile and relaxation functions	High (sufficient basic and clinical observational evidence, well-defined mechanistic pathways)	Indoxyl sulfate (IS), p-cresyl sulfate (PCS)	Medium (in basic and clinical observation phase)
	Markers related to myocardial fibrosis	Reflects the degree of myocardial remodeling, increasing with the progression of chronic kidney disease and accelerating the vicious cycle of cardio–renal crosstalk and aging	High (supported by clinical imaging cohort evidence)	High-sensitivity troponin T, N-terminal pro-B-type natriuretic peptide (NT-proBNP), procollagen type I N-terminal propeptide (P1NP), procollagen type III N-terminal propeptide (P3NP)	Low (used solely as a biomarker for evaluation; no direct therapeutic effect)
Gut–Cardiovascular	Lipopolysaccharide (LPS)	Exacerbation of atherosclerosis	Medium (basic mechanistic research)	LPS, plasmalemma vesicle-associated protein-1 (PV-1), interleukin-1β (IL-1β), IL-6, TNF-α	Low (in basic research stage; no clinical treatment options yet)
	Trimethylamine N-oxide (TMAO)	Endothelial dysfunction	High (robust association between mechanism and clinical outcomes, widely recognized risk target)	TMAO	Medium (in clinical research stage; no treatment regimen available yet)
	Phenylacetylglutamine (PAGln)/phenylacetic acid (PAA)	Cardiac aging	Medium (novel high-quality omics and functional assays for validation)	PAGln, PAA	Low (in basic research stage; no clinical treatment options yet)

## Data Availability

No new data were created or analyzed in this study. Data sharing is not applicable to this article.

## Abbreviations

The following abbreviations are used in this manuscript:

ACEIs	angiotensin-converting enzyme inhibitors
AhR	aryl hydrocarbon receptor
AHA	American Heart Association
AKT	protein kinase B (PKB)
AMPK	AMP-activated protein kinase
Ang II	angiotensin II
APN	adiponectin
ARB	angiotensin receptor blocker
ARNI	angiotensin receptor–neprilysin inhibitor
BMI	body mass index
cGAS	cyclic GMP-AMP synthase
CHF	chronic heart failure
CKD	chronic kidney disease
CKM	cardiovascular-renal-metabolic
CMT	continuous moderate-intensity training
CPT-1	carnitine palmitoyltransferase-1
CVD	cardiovascular disease
DOHaD	developmental origins of health and disease
DPP-4	dipeptidyl peptidase-4
EAA	essential amino acid
EAT	epicardial adipose tissue
ECM	extracellular matrix
eGFR	estimated glomerular filtration rate
ER	endoplasmic reticulum
ERK1/2	extracellular signal-regulated kinase 1/2
EV	extracellular vesicle
FMT	fecal microbiota transplantation
FNDC5	fibronectin type III domain-containing protein 5
FSTL1	follistatin-like protein 1
GFR	glomerular filtration rate
GIP	glucose-dependent insulinotropic polypeptide
GLP-1	glucagon-like peptide-1
GLP-1R	GLP-1 receptor
GLS	global longitudinal strain
GPx	glutathione peroxidase
HF	heart failure
HIF-1	hypoxia-inducible factor-1
HFpEF	heart failure with preserved ejection fraction
HFrEF	heart failure with reduced ejection fraction
HIT	high-intensity interval training
HPA	hypothalamic-pituitary–adrenal
hs-CRP	high-sensitivity C-reactive protein
H_2_S	hydrogen sulfide
IGF-1	insulin-like growth factor-1
IL-1β	interleukin-1β
IL-6	interleukin-6
IS	indoxyl sulfate
LPS	lipopolysaccharide
LVEF	left ventricular ejection fraction
MCP-1	monocyte chemoattractant protein-1
Metrnl	Meteorin-like protein
MGF	mechano-growth factor
MI	myocardial infarction
MRA	mineralocorticoid receptor antagonist
MSTN	myostatin
mtDNA	mitochondrial DNA
mtROS	mitochondrial reactive oxygen species
MuRF1	muscle-specific RING finger protein-1
NF-κB	nuclear factor-κB
NLRP3	NOD-like receptor family pyrin domain containing 3
NRG1	neuregulin-1
NT-proBNP	N-terminal pro-B-type natriuretic peptide
PAA	phenylacetic acid
PAGln	phenylacetylglutamine
PCS	p-cresyl sulfate
P1NP	procollagen type I N-terminal propeptide
P3NP	procollagen type III N-terminal propeptide
PFK-1	phosphofructokinase-1
PGC-1α	peroxisome proliferator-activated receptor γ coactivator-1α
PI3K	phosphatidylinositol 3-kinase
PV-1	plasmalemma vesicle-associated protein-1
RAAS	renin-angiotensin-aldosterone system
RCT	reverse cholesterol transport
RSV	resveratrol
SASP	senescence-associated secretory phenotype
SCFA	short-chain fatty acid
SGLT-2i	sodium-glucose cotransporter 2 inhibitor
SIRT3	sirtuin-3
SNS	sympathetic nervous system
SOD2	superoxide dismutase 2
SPPB	short physical performance battery
STING	stimulator of interferon genes
T2DM	type 2 diabetes mellitus
TGF-β	transforming growth factor-β
TLR4	Toll-like receptor 4
TMAO	trimethylamine N-oxide
TNF-α	tumor necrosis factor-α
VExUS	venous excess ultrasound
XJEK	Xin-Ji-Er-Kang
